# Editorial: Hyperbaric oxygen and the brain

**DOI:** 10.3389/fneur.2022.1078544

**Published:** 2022-12-21

**Authors:** Amir Hadanny, Shai Efrati

**Affiliations:** The Sagol Center for Hyperbaric Medicine and Research, Shamir Medical Center, Ramle, Israel

**Keywords:** hyperbaric oxygen, regenerative medicine, HBOT, TBI, aging, cancer

The central nervous system (CNS) was thought to lack the inherent capability of regeneration following injury, which other tissues in our body possess. The existence of neural stem cells was doubted as well. That gap in understanding our brains' physiology directed researchers and pharmaceutical companies to investigate therapies and drugs that alleviate symptoms rather than focus on the underlying biology. Recent accumulated evidence shows that the existence of adult neuronal stem cells and that brain regeneration is feasible under the appropriate conditions.

Neural stem cells have the potential to generate most, if not all, different types of neurons, and glial cells found in the brain. However, neuronal stem cells are mostly concentrated in the sub-ventricular zone and the hippocampus and the newly generated neuron has to migrate to its target destination and integrate within its new environment. It is estimated that even in healthy brains, only a third of these new neurons reach their destination. This endogenous regeneration response is limited, as migration, integration and survival of these newly generated cells are unsuccessful due to an unfavorable environment.

The use of hyperbaric oxygen therapy (HBOT), harnessing the hyperoxic-hypoxic paradox (HHP), represent one of the first therapeutic intervention already in clinical use today for the specific goal of inducing regeneration of damaged brain tissue.

HHP is a newly suggested paradigm aiming to enhance the endogenous repair mechanisms, while providing an optimal microenvironment. By utilizing repeated intermittent hyperoxic exposures, hypoxia-triggered mediators and cellular mechanisms are paradoxically induced without hypoxia. These effects include stem cell stimulation, migration and differentiation, mitochondrial proliferation/biogenesis, mitochondrial transfer and angiogenesis (Figure 1). Critically, inducing both the hypoxic-induced-factor (HIF) and the vascular endothelial growth factor (VEGF), leads to new blood vessel generation (angiogenesis) under hyperoxia, which provides the required supportive repair environment (Figure 1).

**Figure 1:**
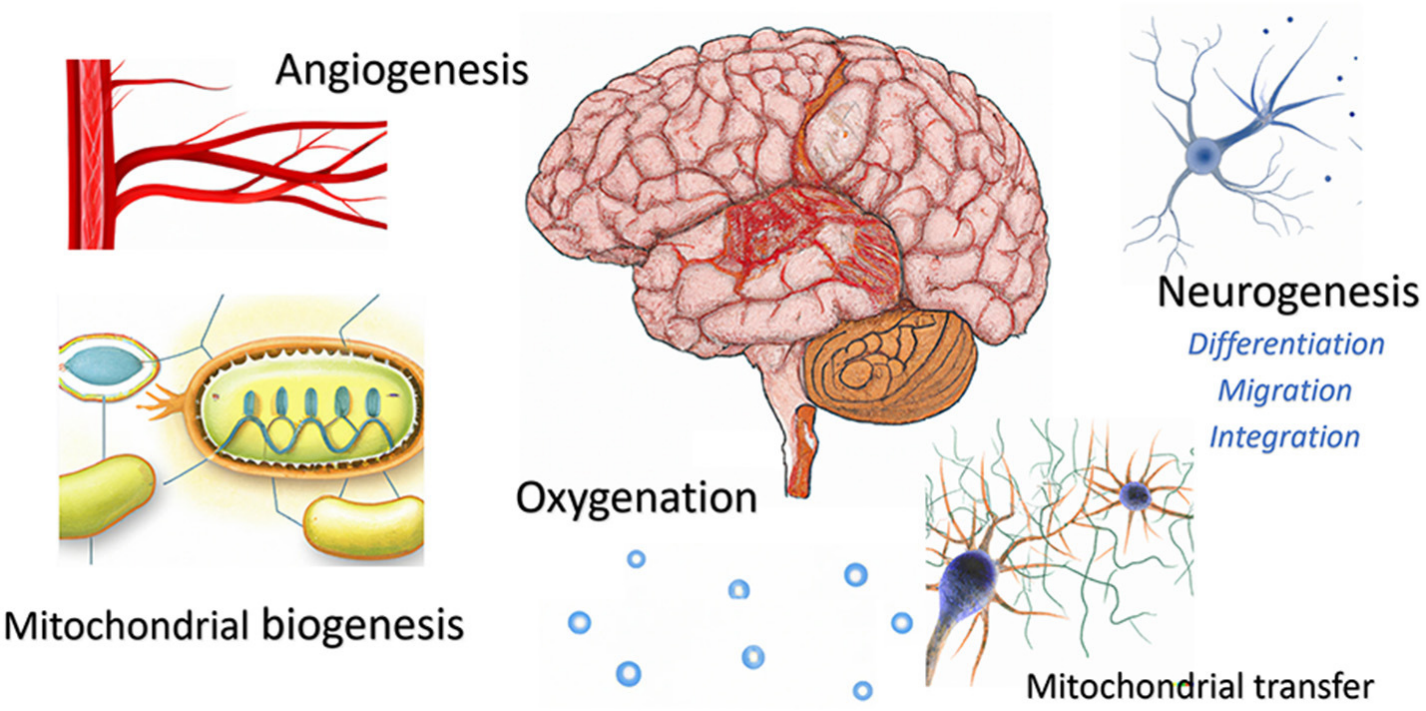
Brain regeneration mechanisms induced by HHP protocols.

The effects of HBOT protocols, harnessing the HHP, in acute traumatic brain injury (TBI) by using HBOT have been demonstrated by many preclinical studies. Human randomized controlled studies showed a significant effect on mortality with an inconclusive effect on neurologic outcome/function due to the significant severity of the patients included in these studies. Chen et al.'s prospective controlled trial on moderate-severe acute TBI patients shows efficacy on neurologic prognosis, cognitive function, injury volume, electrophysiological markers, and biomarkers. With other high quality randomized controlled trials being planned, we expect a revolution in TBI patient-care, including properly designed HBOT protocols.

In the past decade, the treatment of TBI survivors in their chronic stage with HBOT was constantly debated. Major methodological flaws and the lack of patients have failed large scale studies. However, quality data from properly controlled and designed trials in both adults and children suffering from chronic mild TBI, the so-called post-concussion syndrome, demonstrated significant improvements on symptoms, cognition, mood, behavior, and quality of life. However, patient selection based on objective measures remains critical.

During aging, neural stem cells and their progenitors exhibit reduced proliferation and neuron production, which is thought to contribute to age-related cognitive impairment. Just like most body organs in our body, the regenerative capacity in our brain declines after the age of 40 years, so the net balance between regeneration and degeneration leans toward degeneration. Recently, new HBOT protocols, which maximize the HHP effects, have demonstrated brain regeneration, and enhanced cognitive function in the so called normal aging population. Maroon provides a fascinating report documenting o objective outcomes after subjecting himself to a HHP based HBOT protocol. Apart from enhanced cognitive function and brain perfusion, he reports on objective physical performances as well as aging related biomarkers, which can suggest a general regenerative effect of HHP on human aging rather than brain specific effects. These findings are also in accordance with previous results that demonstrate that the same HBOT protocols can induce telomeres elongation and reduce senescent cell counts.

A hypoxic environment also plays a crucial role in cancer development. Due to the uniqueness of the cancer cells' mitochondria, the cancer cells can use anaerobic fermentation of glucose for energy, the so-called Warburg effect. This gives cancer cells a survival advantage over normal healthy cells in an oxygen deprived environment. The mentioned HHP effects on the microenvironment may have an anticancer role, specifically in glioblastoma (GBM) brain tumor. It is well established that solid tumors, including GBM, generate local hypoxia with progressive tissue necrosis, which restrict many of the chemotherapy and radiotherapy effects. By generating new pathways *via* effective angiogenesis, distinguished from the abnormal angiogenesis generated by the tumor, and delivering high levels of oxygen *via* diffusion, HHP based protocols may change the tumor microenvironment and counteract the tumor's hypoxia. HHP may also be synergistic with chemo- and/or radiotherapy. In addition, HHP protocols have been shown to induce mitochondrial transfer between astrocytes to neurons, which may play an additional role in anticancer effects. Costa et al. provide a literature review on the latest emerging data of radiotherapy/chemotherapy combined with HBOT effects in GBM.

HHP protocols are based on repeated hyperoxic exposures which trigger an upsurge in the expression of cell-protective proteins including antioxidative scavengers. In turn, a new homeostasis is formed with a higher level of these proteins, which enhances the cellular tolerance against harmful stimuli. Therefore, HBOT preconditioning protocols have been suggested to provide tissue protective effects in expected ischemia–reperfusion injuries to the brain, skin, spinal cord, liver, kidney, and heart in various animal models. Additionally, several human studies have demonstrated preoperative efficacy prior to different surgeries (coronary artery bypass grafts, abdominoplasty). Ostrowski et al. report on a new possible preconditioning mechanism involving the proteasome.

In summary, the current topic provides a glimpse into the future of HHP based HBOT protocols in neuroscience. We thank the authors for their valuable contributions and efforts, and we encourage additional research groups to further investigate this promising intervention in other clinical settings.

## Author contributions

All authors listed have made a substantial, direct, and intellectual contribution to the work and approved it for publication.

